# African Americans’ Diminished Returns of Parental Education on Adolescents’ Depression and Suicide in the Adolescent Brain Cognitive Development (ABCD) Study

**DOI:** 10.3390/ejihpe10020048

**Published:** 2020-06-16

**Authors:** Shervin Assari, Shanika Boyce, Mohsen Bazargan, Cleopatra H. Caldwell

**Affiliations:** 1 Department of Family Medicine, Charles R Drew University of Medicine and Science, Los Angeles, CA 90059, USA; Mohsenbazargan@cdrewu.edu; 2 Department of Pediatrics, Charles R Drew University of Medicine and Science, Los Angeles, CA 90059, USA; ShanikaBoyce@cdrewu.edu; 3 Department of Family Medicine, University of California, Los Angeles, Los Angeles, CA 90095, USA; 4 Department of Health Behavior and Health Education, School of Public Health, University of Michigan, Ann Arbor, MI 48109, USA; cleoc@umich.edu; 5 Center for Research on Ethnicity, Culture, and Health, School of Public Health, University of Michigan, Ann Arbor, MI 48109, USA

**Keywords:** population groups, racism, social segregation, depressive disorder, major, mood disorders, affect, suicide, attempted, educational status, social class, marital status

## Abstract

To investigate racial and ethnic differences in the protective effects of parental education and marital status against adolescents’ depressed mood and suicidal attempts in the U.S. As proposed by the Marginalization-related Diminished Returns (MDRs), parental education generates fewer tangible outcomes for non-White compared to White families. Our existing knowledge is very limited regarding diminished returns of parental education and marital status on adolescents’ depressed mood and suicidal attempts. To compare racial groups for the effects of parental education and marital status on adolescents’ depressed mood and suicidal attempt. This cross-sectional study included 7076 non-Hispanic White or African American 8-11 years old adolescents from the Adolescent Brain Cognitive Development (ABCD) study. The independent variables were parental education and marital status. The main outcomes were depressed mood and suicidal attempts based on parents’ reports using the Kiddie Schedule for Affective Disorders and Schizophrenia (K-SADS). Age and gender were the covariates. Race was the moderator. Logistic regression was used to analyze the ABCD data. Overall, parental education was associated with lower odds of depressed mood (OR = 0.81; 95% CI = 0.67–0.99; *p* = 0.037) and having married parents was associated with lower odds of suicidal attempts (OR = 0.50; 95% CI = 0.28–0.91; *p* = 0.022). In the pooled sample, we found interaction terms between race with parental education and marital status on the outcomes, suggesting that the protective effect of having married parents against depressed mood (OR = 1.54; 95% CI = 1.00–2.37; *p* = 0.048) and the protective effect of having married parents against suicidal attempts (OR = 6.62; 95% CI = 2.21–19.86; *p* = 0.001) are weaker for African Americans when compared to Whites. The protective effects of parent education and marital status against depressed mood and suicidal attempts are diminished for African American adolescents compared to White adolescents. There is a need for programs and interventions that equalize not only socioeconomic status (SES) but also the marginal returns of SES for racial minority groups. Such efforts require addressing structural and societal barriers that hinder African American families from translating their SES resources and human capital into tangible outcomes. There is a need for studies that can minimize MDRs for African American families, so that every individual and every family can benefit from their resources regardless of their skin color. To achieve such a goal, we need to help middle-class African American families secure tangible outcomes in the presence of SES resources.

## 1. Introduction

In addition to race and ethnicity, socioeconomic status (SES) factors—such as parental education [1] and family structure [2]—are among the major social determinants of early adolescent outcomes. Married and educated families have higher levels of parental involvement, monitoring, and positive parenting that all have positive consequences across several domains of adolescents’ development [3]. Some recent evidence, however, shows that when compared to Whites, African American adolescents show weaker effects of parental education [4] and family structure [5] on tangible adolescents outcomes, a pattern called Minorities’ Diminished Returns (MDRs) [6,7].

Parental education (i.e., highest education level of parents) [8,9,10,11] and family structure (i.e., parental marital status) [2] are two strong predictors of a wide range of positive developmental and health outcomes of adolescents across domains. Studies have shown that highly educated [8,9,10,11] and married [12] families are buffered against economic and non-economic adversities, stress, and risk.

Given the central role of parental education [8,9,10,11] and marital status [2] on adolescents outcomes, and because race/ethnicity has been historically linked to low SES, low education, and unmarried status [13], past research has traditionally attributed some of the racial and ethnic disparities in adolescent outcomes to the lower level of parental education as well as lower likelihood of being from a married family in racial minority families such as African Americans [9,14,15]. These studies have concluded that enhancing education levels of racial minorities and helping them build stable married families would be the main strategy for closing the racial and ethnic gap in the lives of adolescents [16].

As shown by the MDRs literature, however, the education level [8,17] and marital status [5] of parents may generate differential outcomes for diverse racial and ethnic groups. Due to social stratification, discrimination, and structural inequalities, racial and ethnic minorities do not have the same opportunities to navigate the existing resource systems and mobilize their own resources to secure tangible outcomes. As a result, high education level [7,17,18,19] and marital status [2] tend to generate fewer positive health outcomes for African American than for White families. As a result, African American adolescents show worse-than-expected health outcomes due to the weaker than expected effects of parental education and marital status relative to their counterparts [6,7,19,20].

Education level [6,7] and marital status [2] of self and parents also differentially protect African American and White families against mood disorders and suicide. Among adults, education level has smaller protective effects on the risks of depression [21] and suicide attempts [22] for African American people compared to White people. There is even some evidence linking high SES to poor mental health for African American adolescents [23] and adults [21], both in terms of depression [21] and suicidal ideation [22]. Some of the undesired mental health outcomes faced by high SES African American families are attributed to the fact that these families are located at a proximity to Whites, which increases the likelihood of experiencing interpersonal discrimination [24,25]. Needless to say, discrimination is a risk factor of undesired developmental and health outcomes [26,27,28], including depression [29] and suicide [30].

However, for multiple reasons, we need more studies on the MDRs of family resources on depression and suicide in African American adolescents. First of all, there is a dearth of evidence about race and ethnicity as moderating factors for the effects of parental resources on mood and suicide of adolescents. Second, most of the existing literature on MDRs is focused on other outcomes such as adolescents body mass index (BMI) [31], self-rated health (SRH) [20], attention deficit hyperactivity disorder (ADHD) [32], subjective health and well-being [17], impulse control [19], and school attachment [33], education level [34], school performance [1], aggression [4], and substance use [4]. From this literature, very few studies have focused on MDRs on depressed mood and suicidal attempts.

### Aims

To extend the previous knowledge on the MDRs of parental education and marital status on African American adolescents’ mental health, we compared African American and White families for the effects of parental education and marital status on adolescents’ depressed mood and suicidal attempts. Our first hypothesis was that adolescents with high parental education and married parents would have lower odds of depressed mood and suicidal attempts. Our second hypothesis, however, was of weaker effects of parental education and marital status on adolescents’ depressed mood and suicidal attempts for African American and White families.

## 2. Materials and Methods

### 2.1. Design and Settings

This secondary analysis used a cross-sectional design and analyzed the existing wave 1 data of the Adolescent Brain Cognitive Development (ABCD) study [35,36,37,38,39]. ABCD is the largest national, state-of-the-art brain imaging study of adolescents’ brain development in the history of United States. The advantages of the ABCD study include (1) the large sample size, (2) the national sample, (3) the large sample of African Americans, (4) publicly available data, (5) considerable socioeconomic variables, and (6) vast behavioral measures [35,36,37,38,39]. For more detailed information about ABCD’s purpose, methodology, and measurement, please see the ABCD methods papers [35,40].

### 2.2. Sampling

The ABCD sample were selected across 21 sites located within multiple cities in the United States. This sample was recruited through schools [41]. Although the study sampling design had major departures from a multi-stage probability sample design, the ABCD study is believed to generate a nationally representative sample of adolescents. The sample was recruited by a nationally distributed set of 21 study sites. These sites then used a probability sampling of schools within their defined catchment areas and recruitment of eligible children from sample school. The mentioned departure from the traditional probability sampling of U.S. children was selection of the participating neuroimaging sites. They were chosen non-randomly, based on the quality of their application to the NIH. Still, the 21 ABCD study sites are dispersed (nationally), and are believed to generate a nationally representative sample with high diversity [41].

### 2.3. Analytical Sample

For this analysis, 7076 8–11 years old non-Hispanic White or African American adolescents who had data on K-SADS with past depressed mood were included. From this number, only 3271 adolescents had data on suicidal attempts, so our sample size was only 3271 for our analysis of suicidal attempts. Our descriptive statistics are for the 7076 individuals who entered our analysis. Our study did not include people from the Hispanic ethnic background.

### 2.4. Study Variables

The study variables included data on sociodemographic variables as well as previous history of depressed mood and suicidal attempts.

#### 2.4.1. Outcome

**Depressed mood**. Depressed mood, the first main outcome in this study, was measured using the Kiddie Schedule for Affective Disorders and Schizophrenia (K-SADS) [42]. Parents were interviewed and reported the symptoms. Depressed mood in the past, regardless of its timing, was the outcome. This variable was treated as a categorical/dichotomous outcome, with 0 reflecting no and 1 reflecting depressed mood.

**Suicidal attempts**. Suicidal attempts, the second main outcome in this study, were also measured using the K-SADS [42]. Parents were interviewed and reported the symptoms. Any suicidal attempts in the past, regardless of their timing, were the outcome. This variable was treated as a categorical/dichotomous outcome with 0 for absence and 1 for presence of ay suicidal attempts.

#### 2.4.2. Moderator

**Race.** In the ABCD study, race, a self-identified variable, was a dichotomous variable (African Americans = 1, Whites = 0). The moderator was defined based on Baron and Kenny, and was a variable that changed the magnitude of the direction of an association of interest [43,44].

#### 2.4.3. Independent Variable

**Parental Education**. Parents were asked, “What is the highest grade or level of school you have completed or the highest degree you have received?” This variable was treated in the current study as individuals with high school graduation, General Educational Development (GED) or equivalent Diploma as 1 and any education level less than that as 0. Thus this 0/1 variable was coded 0 for low and 1 for high parental education.

*Parental Marital Status.* Parental marital status was self-reported by the parents. This variable was a dichotomous variable coded as married = 1 and other = 0.

#### 2.4.4. Confounders

**Age and Gender**. Parents reported the age of their adolescent children. Age was a continuous measure in years. Gender was a dichotomous variable: males = 1 and females = 0.

### 2.5. Data Analysis

For our data analyses, we applied SPSS. Descriptive data were summarized using means, standard deviations (SD), frequency, and relative frequency (%). To perform bivariate analyses, we used independent sample t-test and Chi-square. To perform our multivariable analyses, we performed four logistic regression models for each outcome. Logistic regression is commonly used for biomedical as well as clinical and sociological, public health, and social science research [45]. The first two models were estimated in the overall sample that included White and African Americans. These models controlled for gender as well as age. *Model 1* (Main Effect Model) was performed with race, parental education, marital status, and covariates. *Model 2* (Interaction Model) included the main effects of race, parental education, marital status, covariates, as well as two multiplicative/interaction terms parental education and marital status and race. Model 3 was performed in Whites and Model 4 was performed in African Americans. In all these models, either depressed mood or suicidal attempts was the outcome. Odds Ratio (OR), S.E., 95% CI, and p-value were reported for each model. A p-value equal to or smaller than 0.05 was considered significant.

### 2.6. Ethical Aspect

The mother study, the ABCD study, received Institutional Review Board (IRB) approval by the University of California, San Diego (UCSD). All adolescent participants provided assent. Their parents provided consent [40]. We received the ABCD data through an agreement between Charles R. Drew University and NIH/NDA. As the ABCD data that we received were fully de-identified, our study was considered as non-human subject research, thus was exempted from a full review.

## 3. Results

### 3.1. Descriptives

This analysis included a total sample of 7076 pre-adolescents. All participants were between 8 and 11 years old, and were either African American (n = 1929; 27.3%) or White (n = 5147; 72.7%). Table 1 presents descriptive statistics of the pooled sample (Table 1).

### 3.2. Descriptives

Table 1 also compares Whites and African Americans for the study variables. As shown by this table, Whites and African Americans did not differ in age or gender but differed in parental education and marital status of the family, as well as depressed mood and suicidal attempts. Compared to Whites, African Americans had lower parental education and were less likely to be from married families; however, they were more likely to have depressed mood and suicidal attempts compared to Whites.

### 3.3. Multivariate Analysis in the Overall Sample

Table 2 shows the results of our logistic regressions in the overall sample. We reported the results of two models for each outcome. *Model 1* (Main Effect Model) revealed a protective effect of parental education on experiencing depressed mood, but not for having married parents (OR = 0.81; 95% CI = 0.67-0.99; *p* = 0.037). It also showed that there is a protective effect of having married parents against suicidal attempts, but not for education (OR = 0.50; 95% CI = 0.28-0.91; *p* = 0.022).

*Model 2* (Interaction Model) showed interaction terms between race with parental education and marital status on the outcomes, suggesting that the protective effects of having married parents against depressed mood (OR = 1.54; 95% CI = 1.00-2.37; *p* = 0.048) is weaker for African American adolescents relative to their White counterparts. *Model 2* also showed an interaction term between race with parental education on suicidal attempts (OR = 6.62; 95% CI = 2.21-19.86; *p* =0.001), meaning that the protective effect of having married parents is weaker for African American adolescents relative to their White counterparts (Table 2).

### 3.4. Multivariate Analysis by Race

Table 3 shows the results of our logistic regression models specific to racial groups. Again, similar models were performed for each outcome. *Model 3* (Whites) showed the protective effects of parental education on depressed mood (OR = 0.77, 95% CI = 0.61-0.96; *p* = 0.021) and suicidal attempts (OR = 0.29; 95% CI = 0.14-0.61, *p* = 0.001) in Whites; however, the same pattern was absent for both depressed mood (OR = 0.92, 95% CI = 0.63-1.34; *p* = 0.678) and suicidal attempts (OR = 1.86; 95% CI = 0.83-4.16, *p* = 0.132) in *Model 4* for African Americans (Table 3).

## 4. Discussion

Overall, there were protective effects of high parental education and having married parents against depressed mood and suicidal attempts in the U.S. These protective effects, however, were dependent upon race. Higher parental education and marital status were linked to lower odds of adolescents’ depressed mood and suicidal attempts for White but not African American adolescents. That means African American adolescents have depressed mood and suicidal attempts across all levels of parental education and marital status.

Regarding our first finding, research has previously shown that adolescents from highly educated families do drastically better regarding depressed mood and suicidal attempts. Adolescents from high SES families have a lower risk of depression [23], anxiety [5], aggression [4], and substance use [4]. Family SES is a fundamental cause of health and illness [46,47]. Similarly, adolescents’ outcomes show a social gradient follows the availability of SES resources [48,49,50]. High parental education may better generate adolescents’ outcomes. This study showed lower depressed mood and suicidal attempts in adolescents with parents who have high levels of education. The universal nature of the protection of parental education is in line with the root cause, fundamental cause theory. Given that early outcomes in adolescents are the drivers of future outcomes during adulthood [51], the diminished effects of parental education on adolescents’ mood and suicide should be taken seriously.

Our second finding was also a successful replication of some previous work on MDRs. In a study of a clinical sample, high education reduced the suicidal risk of White but not African American adults [52]. In two other studies, high education and income showed boosting effects on happiness, subjective mental health, and positive emotions for White but not African American adolescents [53]. In multiple other studies, SES was reported to be a risk factor, not a protective factor, for depression in African Americans—particularly boys and men [23,29]. Hudson has published several papers on this pattern [54,55,56,57,58].

There is a growing literature on the diminished returns of parental education and parental marital status on adolescents’ outcomes. This literature shows that African American individuals and families show weaker effects of family SES on health outcomes compared to their White counterparts [18,59]. MDRs within families contribute to the trans-generational effects of inequalities from one generation to another. MDRs are robust and hold for various SES resources, age groups, outcomes, and marginalization types [6,7].

MDRs have also been reported for other domains. In a recent study, MDRs of education was found for all subdomains of the Child Behavior Checklist (CBCL) [60]. African American adolescents receive smaller academic benefits from their parental education than their white counterparts [61,62]. In another study, MDRs were found for inhibitory control [63], impulsivity [19], aggression [4], school performance [64], depression [29], and anxiety [5].

Several mechanisms may explain why SES indicators such as parental education and marital status have weaker protective effects for African American families compared to Whites [18,65]. One mechanism is labor market discrimination, that influences highly educated African American parents. Due to the existing labor market dual system, highly educated African American parents work in worse jobs and conditions compared to Whites [18,65,66]. In the U.S., racism influences all U.S. institutions, and the labor market is not an exception to this rule. Thus, similarly educated African American families make less income and report higher levels of stress than White families [8]. Upward social mobility is also more stressful and taxing for African American individuals than White individuals [6,7]. Historically, African Americans have had less political power. Thus, they have not been able to influence laws and policies [67,68,69]. In the absence of a strong political presence from minority groups, U.S. policies have enhanced the benefits of the dominant group, Whites. In these processes, African American communities are left poor, so they struggle with many societal barriers on a daily basis [6,7]. For their upward social mobility, they face an uphill battle, and use effortful coping, which is predictive of anxiety and depression [57,70,71,72]. This specific type of high-cost psychologic coping is called John Henryism [70,73,74,75,76,77]. Hudson, James, and others have described these processes and their implications for health in detail [57,74,75,78,79].

At high levels of educational attainment, African American families and parents face a disproportionately higher level of stress in their daily lives. Such an increased exposure and vulnerability to general, economic, and race-related stress may reduce how families can absorb the health effects of their education [65]. Highly educated African American families may experience more, not less, discrimination on a daily basis [24,25,55,57,58,80]. Highly educated African Americans may show heightened sensitivity and vulnerability to the negative consequences of interpersonal discrimination [53].

Racial and ethnic gaps in the marginal returns of parental education (i.e., MDRs) may be due to worse education quality in urban and public schools where most African American communities live and receive their education. African Americans and Whites attend schools that are separate and unequal in terms of quality and resources. On top of this racial inequality in access to the opportunity structure [18,81], African American adolescents experience high levels of discrimination from their teachers [64,82]. Thus, African Americans receive minimal chances to effectively mobilize their education and secure outcomes [6,7].

Environmental and contextual factors, such as residential segregation, also explain the MDRs. Due to residential and school segregation, students from high SES families may still be sent to poor schools. Such schools have less funding, have high-risk peers, and a high level of social and environmental risk [83]. In contrast to high SES African American families, high SES White families send their adolescents to predominantly White schools that have more funding, higher budgets, and higher-quality teachers [84,85]. Thus, not only SES, but also race, affects school options for adolescents from high SES African American families, unless a high SES family has left the community and moved to a White neighborhood [62,83].

These results are important because they show that, as documented by the MDRs framework, even the very same SES resources generate unequal outcomes, with racial minority groups being at a relative disadvantage when compared to the socially privileged groups such as Whites [6,7]. While this study exclusively documented the MDRs of parental education and marital status, MDRs patterns seem to be independent of SES resource, outcome, and even marginalization type [6,7].

The study presented here failed to explore the contextual and societal factors and processes that may cause intergenerational MDRs for racial and ethnic minority families. MDRs have been in part attributed to institutional, structural, and multilevel racism [6,86]. African American individuals are likely to stay in poor neighborhoods despite high SES. As a result, adolescents from African American families are more likely to remain at risk of environmental exposures. Similarly, adolescents from highly educated African American families may remain at risk of interacting with high-risk peers who are involved in behavioral problems [4,87].

## 5. Limitations

This study had a few methodological drawbacks and limitations. One limitation of the current study included a cross-sectional design that limits any inference of causal associations between race, parental education, marital status, and adolescents’ depression and suicide. Similar to most of the rest of the MDRs literature, this study focused exclusively on African American and White families. We still need more studies on other sub-groups of the society, which include various marginalizing social identities other than race and ethnicity. Not only race and ethnicity, but also many other marginalizing social identities—such as immigration—may reduce the gains that are expected to follow parental education and marital status [59,88,89,90]. Similarly, this study only investigated the MDRs of parental education and family structure. This analysis has only few independent variables. Although this is the limitation of the data and not of the analysis itself, it would be reasonable to expect that the results would be different if more explanatory variables were included. For example, the data do not include information on income and occupational status of parents, two important indicators of SES. Research is still needed on the MDRs of other SES indicators such as wealth, income, and parental employment. MDRs may also be relevant to non-economic resources such as psychological assets [91,92]. Future research should also test if neighborhood income or racial composition can explain the MDRs of parental education and family structure for African American adolescents and families. It is still unknown which contextual factor mediates (explain) or moderate (undo) the observed MDRs of parental education and marital status on African America adolescents’ depression and suicide.

## 6. Conclusions

Compared to Whites, African American adolescents remain at high risk of depressed mood and suicidal attempts, despite high parental education and having married parents. The benefits of parental education and family structure on adolescents’ depressed mood and suicidal attempts are not equally received by racial groups. Societal and structural factors such as segregation, racism, social stratification, and pervasive discrimination limit African American families’ abilities to leverage their resources, including education. Clinicians, policymakers, and researchers should all be aware that parental education and marital status consistently generate less measurable desired outcomes for privileged groups in comparison to disfranchised groups.

## Figures and Tables

**Table 1:** Descriptive data overall.

	Whites (*n* = 5147)		African Americans (*n* = 1929)		All (*n* = 7076)	
	n	%	n	%	n	%
**Race**						
Whites	5147	100.0	5147	72.7	-	-
African Americans	-	-	1929	27.3	1929	100.0
**Gender**						
Male	2423	47.1	3381	47.8	958	49.7
Female	2724	52.9	3695	52.2	971	50.3
Marital Sttaus * ^a^						
Other	1573	30.6	3024	42.7	1451	75.2
Married	3574	69.4	4052	57.3	478	24.8
Parental Education * ^a^						
Did not complete highschool	896	17.4	2217	31.3	1321	68.5
Completed highschool	4251	82.6	4859	68.7	608	31.5
Depressed Mood * ^a^						
No	4774	92.8	6517	92.1	1743	90.4
Yes	373	7.2	559	7.9	186	9.6
Suicidal Attempt * ^a^						
No	2485	98.7	3208	98.1	723	96.0
Yes	33	1.3	63	1.9	30	4.0
	**Mean**	**SD**	**Mean**	**SD**	**Mean**	**SD**
**Age**	9.47	0.50	9.47	0.51	9.46	0.51

Note: * *p* < 0.05 for comparison of Whites and African Americans. ^a^ Chi square test; SD = Standard Deviation; Sample size is 3271 for suicidal attempts.

**Table 2:** Summary of logistic regressions overall.

	Model 1 (Main Effects)			Model 2 (Interaction Effects)		
	OR	95% CI	P	OR	95% CI	P
**Depressed Mood (n = 7076)**						
Race (African American)	1.13	0.91–1.41	0.273	0.86	0.63–1.17	0.340
Gender (Male)	0.88	0.74–1.05	0.149	0.88	0.74–1.05	0.144
Age	1.34 *	1.13–1.58	0.001	1.33 *	1.12–1.58	0.001
Parental Education (Completed High School)	0.81 *	0.67–0.99	0.037	0.77 *	0.62–0.97	0.024
Marital Status (Married)	0.83	0.67–1.03	0.091	0.70 *	0.54–0.91	0.007
Marital Status (Married) × Race	-	-	-	1.54 *	1.00–2.37	0.048
Parental Education × Race	-	-	-	1.20	0.77–1.86	0.413
Constant	0.01 *		0.000	0.01 *		0.000
**Suicidal Attempt (n = 3271)**						
Race (African American)	1.91 *	1.06–3.43	0.030	0.74	0.36–1.55	0.431
Gender (Male)	1.23	0.74–2.04	0.431	1.24	0.74–2.06	0.412
Age	1.09	0.66–1.80	0.728	1.11	0.67–1.83	0.681
Parental Education (Completed High School)	0.63	0.35–1.13	0.120	0.29 *	0.13–0.60	0.001
Marital Status (Married)	0.50 *	0.28–0.91	0.022	0.36 *	0.18–0.74	0.005
Parental Education × Race	-	-	-	6.62 *	2.21–19.86	0.001
Marital Status (Married) × Race	-	-	-	1.96	0.65–5.86	0.231
Constant	0.01		0.066	0.02		0.094

Note: OR = Odds Ratio; CI = Confidence Interval; * *p* < 0.05.

**Table 3:** Summary of logistic regressions by race.

	Model 3 (Whites)			Model 4 (African Americans)		
	OR	95% CI	P	OR	95% CI	P
**Depressed Mood (n = 7076)**						
Gender (Male)	0.81 *	0.65–1.00	0.047	1.04	0.77–1.41	0.780
Age	1.23 *	1.00–1.52	0.050	1.56 *	1.16–2.09	0.003
Parental Education (Completed High School)	0.77 *	0.61–0.96	0.021	0.92	0.63–1.34	0.678
Marital Status (Married)	0.70 *	0.54–0.91	0.008	1.08	0.76–1.52	0.674
Constant	0.02 *		0.000	0.00 *		0.000
**Suicidal Attempt (n = 3271)**						
Gender (Male)	1.49	0.73–3.06	0.276	1.01	0.49–2.11	0.968
Age	1.09	0.55–2.16	0.810	1.13	0.54–2.36	0.743
Parental Education (Completed High School)	0.29 *	0.14–0.61	0.001	1.86	0.83–4.16	0.132
Marital Status (Married)	0.36 *	0.18–0.74	0.005	0.72	0.31–1.65	0.432
Constant	0.02		0.229	0.01		0.214

Note: OR = Odds Ratio; CI = Confidence Interval; * *p* < 0.05.
